# Investigating the Reliability of Novel Nasal Anthropometry Using Advanced Three-Dimensional Digital Stereophotogrammetry

**DOI:** 10.3390/jpm12010060

**Published:** 2022-01-06

**Authors:** Zhouxiao Li, Yimin Liang, Thilo Ludwig Schenck, Konstantin Frank, Riccardo Enzo Giunta, Konstantin Christoph Koban

**Affiliations:** 1 Division of Hand, Plastic and Aesthetic Surgery, University Hospital, LMU, 80336 Munich, Germany; thilo.schenck@hotmail.de (T.L.S.); konstantin.frank@med.uni-muenchen.de (K.F.); riccardo.giunta@med.uni-muenchen.de (R.E.G.); konstantin.koban@med.uni-muenchen.de (K.C.K.); 2 Department of Plastic and Reconstructive Surgery, Shanghai 9th People’s Hospital, Shanghai Jiao Tong University School of Medicine, Shanghai 200086, China; acho_liang@hotmail.com

**Keywords:** 3D surface imaging, anthropometry, nasal morphology, reliability

## Abstract

Three-dimensional surface imaging systems (3DSI) provide an effective and applicable approach for the quantification of facial morphology. Several researchers have implemented 3D techniques for nasal anthropometry; however, they only included limited classic nasal facial landmarks and parameters. In our clinical routines, we have identified a considerable number of novel facial landmarks and nasal anthropometric parameters, which could be of great benefit to personalized rhinoplasty. Our aim is to verify their reliability, thus laying the foundation for the comprehensive application of 3DSI in personalized rhinoplasty. We determined 46 facial landmarks and 57 anthropometric parameters. A total of 110 volunteers were recruited, and the intra-assessor, inter-assessor, and intra-method reliability of nasal anthropometry were assessed through 3DSI. Our results displayed the high intra-assessor reliability of MAD (0.012–0.29, 0.003–0.758 mm), REM (0.008–1.958%), TEM (0–0.06), rTEM (0.001–0.155%), and ICC (0.77–0.995); inter-assessor reliability of 0.216–1.476, 0.003–2.013 mm; 0.01–7.552%, 0–0.161, and 0.001–1.481%, 0.732–0.985, respectively; and intra-method reliability of 0.006–0.598°, 0–0.379 mm; 0 0.984%, 0–0.047, and 0–0.078%, 0.996–0.998, respectively. This study provides conclusive evidence for the high reliability of novel facial landmarks and anthropometric parameters for comprehensive nasal measurements using the 3DSI system. Considering this, the proposed landmarks and parameters could be widely used for digital planning and evaluation in personalized rhinoplasty, otorhinolaryngology, and oral and maxillofacial surgery.

## 1. Introduction

Objective and comprehensive craniofacial soft tissue measurements provide a quantitative basis for surgeons’ consultation, as well as for preoperative and post-operative outcome comparison and follow-up. Advances in craniofacial anthropometry in the last few decades have combined the objective, yet mostly two-dimensional (2D), common and direct anthropometric examinations with tape measure, caliper, and angular measurement, as well as subjective 2D photography. Moreover, 2D photogrammetry has been widely applied to evaluate rhinoplasty outcomes such as nasal tip position, nasal alare width, and nostril shape, as well as in nasal analysis in ethnic groups [1,2]. However, a significant amount of time is required to record the complexity of the nose in detail [3,4,5].

Compared to direct measurements, 3DSI offers more detailed and extensive measurements, including distance, curvature, volume, angle, and surface area [6,7]. With the rise of 3DSI in the last decade, there have been some studies on its application for nasal anthropometry. Their findings have shown considerable reliability and feasibility in the planning and follow-up of rhinoplasty and craniomaxillofacial surgery. Nevertheless, these reports have significant limitations. Firstly, the diverse demographics of the subjects suggest that they are insufficient for stratification [8,9]. In addition, the 3DSI devices involved in these studies are technically obsolete and lack supporting analysis software for data computation. The 3D models need to be exported to third-party software, which increases systematic errors [9,10]. Furthermore, although some scholars have compared 3DSI measurements with direct measurements using tape and calipers, there is little discussion of the errors and biases generated within 3DSI and the errors in the placement of landmarks by different assessors, which is not adequate to prove the reliability of 3DSI in nasal anthropometry [10,11]. Most importantly, only a very limited number of facial landmarks and nasal anthropometric parameters were included in these studies, which limits their relevance to clinical practice [8,9,10,11].

For the mentioned reasons, we have introduced and implemented a considerable number of novel nasal landmarks into an established portfolio of 3D derived landmarks, as well as more comprehensive and original nasal anthropometric parameters, including angles, distances, and ratios, to ensure standardized and accurate coverage of the nasal region. There is no evidence to indicate whether different users and 3D image capture sessions produce consistent measurements. Therefore, the reliability of these nasal landmarks and parameters should be rigorously validated before their broad application in clinical practice. Our study focused on validation of the reliability and consistency of these novel nasal landmarks and anthropometric parameters using a new generation of 3DSI system, the latest Vectra XT-based 3D technology and matched specialized 3D medical measurement software developed by Canfield Inc. (Parsippany, NJ, USA), to depict the 3D information of nasal anthropometry and to provide objective and personalized stereo instructions for related clinical consultation.

## 2. Materials and Methods

### 2.1. Volunteers and Recruitment

A total of 110 healthy Caucasian volunteers (55 males and 55 females) between 18 and 65 years were enrolled in this study (Table 1). Each participant gave written informed consent before enrolment. Exclusion criteria were facial malformations, former maxillofacial surgery, and volunteers diagnosed with epilepsy or other seizure disorders. The study was performed in line with the Declaration of Helsinki and was approved by the local university’s ethical committee (REF: 266-13).

### 2.2. 3D Surface Imaging Device (3DSI)

The Vectra XT 3DSI System (Canfield Inc., Parsippany, NJ, USA) is a three-pod passive stereophotogrammetry system with six cameras at a fixed position, specially created for the healthcare sector. The cameras simultaneously capture all images in 3.5 ms, which limits the risk of motion artefacts. It is designed specifically for medicine and can build a 3D model with a 360-degree view from every angle, and various forms of treatment or before-and-after comparisons can be evaluated in this way. Its high reliability for intraoperative facial imaging and measuring facial volume changes has been validated in our previous research reports [6,7]. Based on its widespread use and former validation, it served as the 3DSI in our current study [4,6,7,11,12].

### 2.3. Image Acquisition

In this study, 3DSI was performed for each session with Vectra XT consecutively. Subjects had any jewelry and hair removed from the face, forehead, and ears to fully expose the area to be scanned. Male volunteers were asked to shave, as hair is a major limitation for 3D imaging. All volunteers were asked to keep their mouth closed, without clenching the teeth, and remain in a relaxed, neutral facial expression in the same chair with a fixed backrest. They had to adopt an upright, non-excessive sitting position, and close their eyes. In our consultation rooms, the lighting conditions and background were not specifically altered, so as to achieve conditions similar to our daily lives. Each assessor underwent a separate 3D scan session.

### 2.4. Data Evaluation

**Figure 1:**
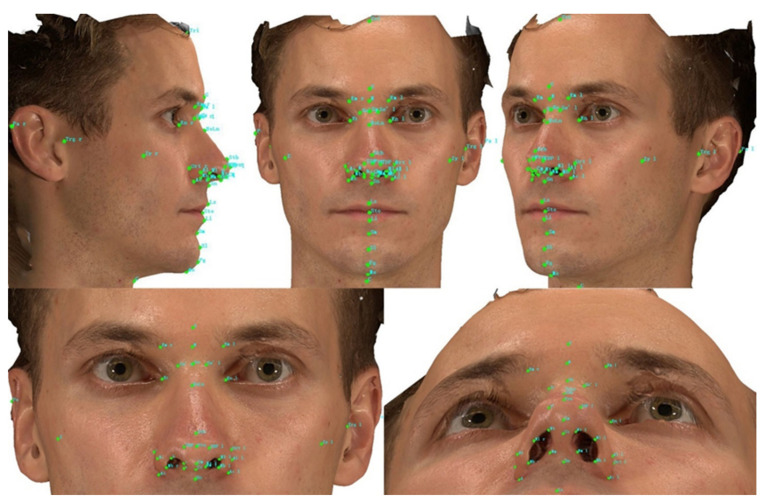
Illustration of the landmarks used in this study (holistic view and local perspective).

**Figure 2:**
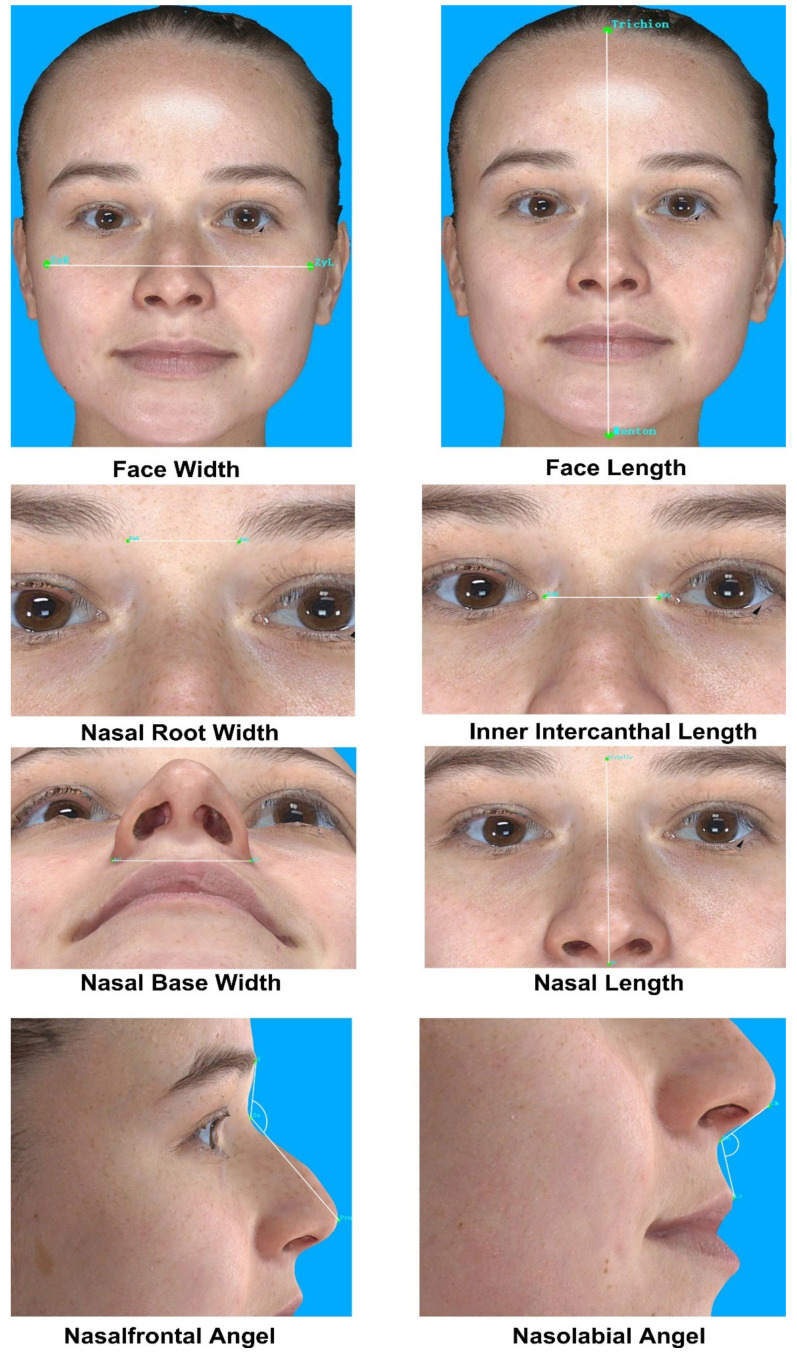
Classic parameters.

**Figure 3:**
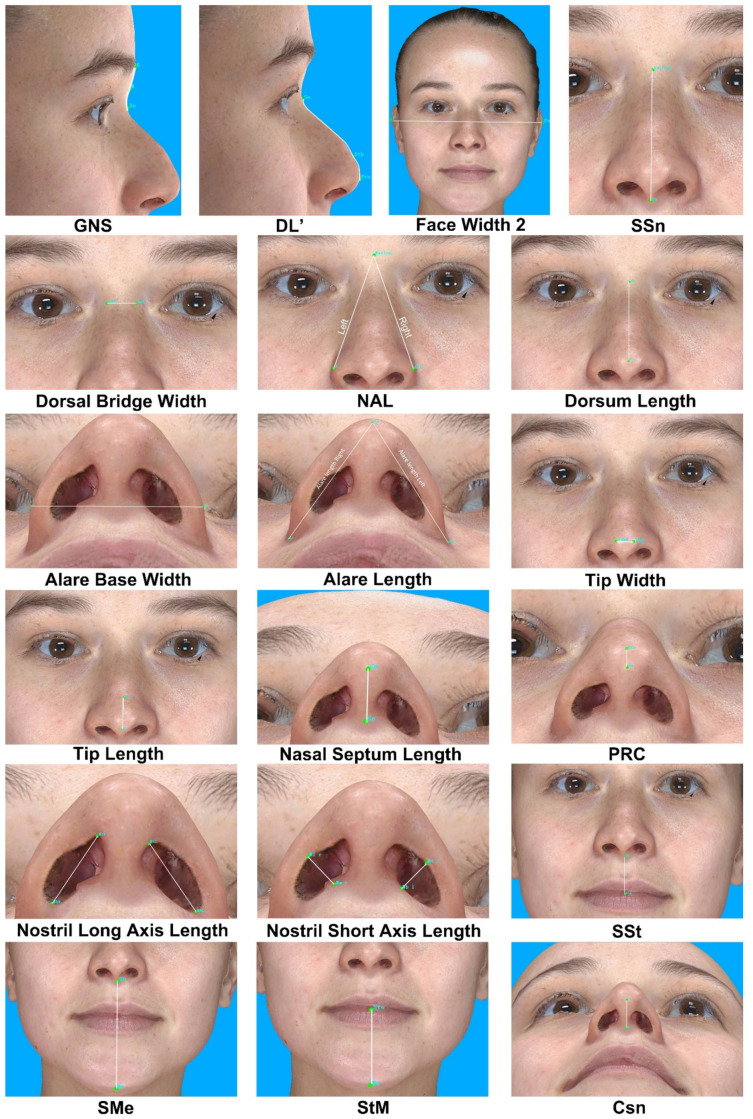
The linear measurements of novel parameters.

**Figure 4:**
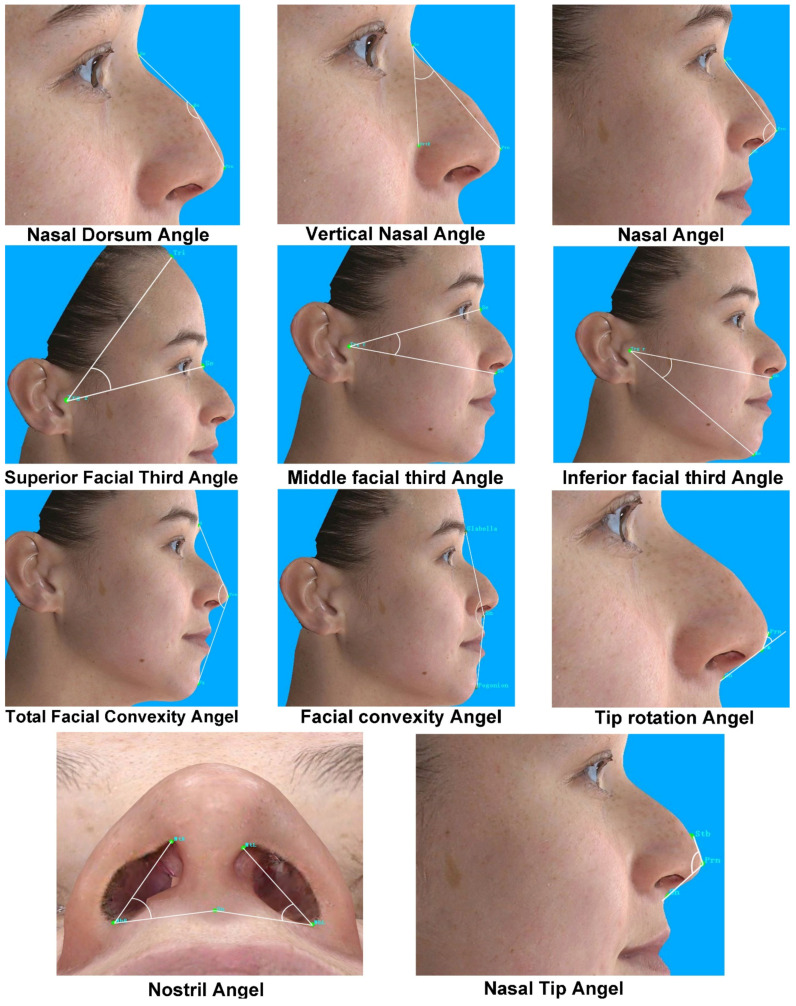
The angular measurements of novel parameters.

A variety of anatomical landmarks and clinical measurements can be performed during the clinical routine of facial surgery. Surgeons used validated anatomical landmarks to obtain a wide range of 2D and 3D measurements. In this study, for each subject, 19 novel landmarks and 27 classic standardized facial landmarks (Figure 1 and Table 2) were placed using the system supportive software Mirror (Canfield Scientific; NJ, USA). The duplicability of landmarks’ designation, landmark positioning, and the data collection procedure was based on Farkas, former related studies, and the cephalometry literature [13,14,15,16]. Subsequently, 49 novel anthropometric parameters and 8 classic parameters were defined. Among them, the parameters were divided into four types of measurements: 2 surface linear distances, 27 projective linear distances, 18 angels, and 10 ratios. Table 3 and Figure 2, Figure 3 and Figure 4 show the composition and visualization of these measurements.

### 2.5. Statistical Analysis

Five statistical indices were calculated to assess intra- and inter-assessor reliability (Table 4). The intraclass correlation coefficient (ICC) indicates high reliability when the value is close to 1 and low reliability when the value is close to 0. Four classes of ICC were defined according to consensus: <0.5, poor; 0.5 to 0.75, moderate reliability; 0.75 to 0.9, good reliability, and ≥0.9, excellent [17,18]. 

Mean absolute difference (MAD) is expressed as the absolute value of the difference between the average value of each variable between two measurements. Technical error of measurement (TEM) is the square root of the variance of measurement error and is calculated as listed in Table 4 [18]. 

As the magnitudes of MAD and TEM were highly positively correlated with the magnitude of the measurements, we combined relative error measurement (REM) and relative TEM (rTEM) to compare the measurement bias of different variables. REM provides an estimate of diversity relative to the magnitude of the measurement, and rTEM reflects bias. REM and rTEM were calculated by dividing the MAD and TEM by the grand mean of the target variables, then multiplying by 100 [8]. Based on the classification criteria proposed in previous research, REM was classified into five levels: <1%, excellent; 1–3.9%, very good; 4–6.9%, good; 7–9.9%, moderate; and >10%, poor [19,20]. The range of excellence for intra-examiner rTEM was <1.5% and inter-examiner <2.0% [21].

Statistical analyses were performed using SPSS Statistics 23.00 (IBM, Armonk, NY, USA). Data normality was tested using the Kolmogorov–Smirnov test for all measurements and all the results were consistent with a normal distribution. We used the GraphPad Prism 8 (GraphPad Software Inc., San Diego, CA, USA) to depict the figures. A difference was considered statistically significant at a probability level of ≤0.05 to guide conclusions.

### 2.6. Ethical Approval

Written informed consent for participation in this study was obtained from all participants following the Declaration of Helsinki protocols (1996). This study was conducted in accordance with regional legislation and good clinical practice (1996) and with approval from the Ethics Committee of the Ludwig Maximilians University of Munich (REF: 266-13).

## 3. Results

### 3.1. Overall

The age of the study participants ranged from 18 to 65 years. Among the volunteers we recruited, there was no statistical difference in age between males (42.23 ± 8.31 years old) and females (40.53 ± 7.99 years old). Supplementary Table S1 show the descriptive statistics (mean and standard error, SD) for classic and novel parameters of intra- and inter-assessor as well as the corresponding *p*-values; the *p* value with an asterisk is less than 0.05, which is statistically significant. The measurements were categorized into four types (surface distance, linear distance, angle, and ratio). The intra-assessor, inter-assessor, and intra-method reliability are described in the following.

### 3.2. Intra-Assessor Reliability with 3D Images

For classical measurements, all eight parameters showed excellent reliability, with an ICC above 0.9. The MAD of almost all linear distances was less than 0.3 mm; only the magnitude for face width (FW) reached 0.758 mm. The angle parameters’ MAD were 0.044 and 0.29 degrees, respectively. 

**Figure 5:**
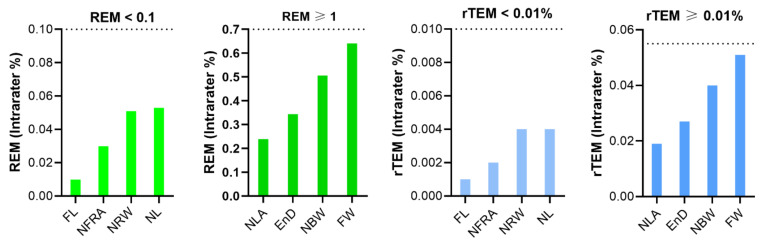
Intra-assessor relative error measurement (REM) and relative technical error of measurement (rTEM) of classical nasal measurements on three-dimensional images.

The REM of all classical parameters were less than 1% and the rTEM of all classical parameters showed very good reliability (Table 5, Figure 5).

All 49 novel measurements displayed good reliability, with an ICC above 0.75 (Table 6). Moreover, 33 measurements showed excellent reliability, with an ICC larger than or equal to 0.9. TFCA had the highest ICC at 1 and the lowest NOAI at 0.77. The MAD of the surface distances GNS and DSL were 0.1 and 0.216 mm, respectively. For most linear distances, the MAD were less than 0.3 mm, except 0.335 mm for alare length left (ALLl). All the angles’ MAD were less than 0.3 degrees and MAD of ratio measurements were less than 0.01, except for nostril aspect ratio left (NARl, 0.013).

**Figure 6:**
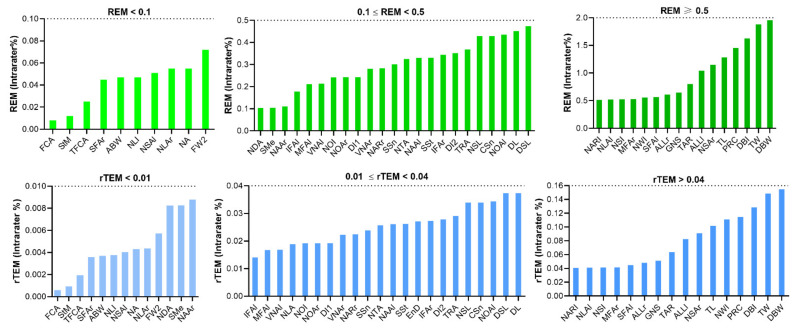
Intra-assessor relative error measurement (REM) and relative technical error of measurement (rTEM) of novel nasal measurements on three-dimensional images.

For REM, 42 parameters were less than 1% and the remaining seven measurements were between 1 and 2%. Among them, the DBW showed the highest REM, with 1.958%. Furthermore, the rTEM of all novel measurements showed very good reliability (Table 6, Figure 6).

### 3.3. Inter-Assessor Reliability with 3D Images

**Figure 7:**
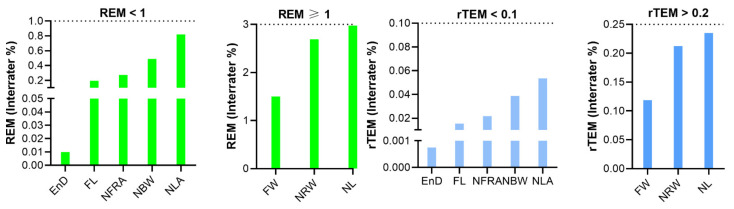
Inter-assessor relative error measurement (REM) and relative technical error of measurement (rTEM) of classic nasal measurements on three-dimensional images.

For classical measurements, the inter-assessor ICC of all eight parameters fell into the good reliability category, with values greater than 0.81. The MAD of angular measurements were less than 1 degree. For linear distance, the MAD of six parameters were less than 1 mm, except for face width (FW) and nasal length (NL), which showed MAD of 1.791 and 2.013 mm, respectively. Five parameters had an excellent REM of less than 1% and the remaining three parameters (FW, NRW, NL) had a REM of between 1 and 3.9% (very good). The rTEM of all classical parameters were less than 0.25% (excellent) (Table 5, Figure 7).

**Figure 8:**
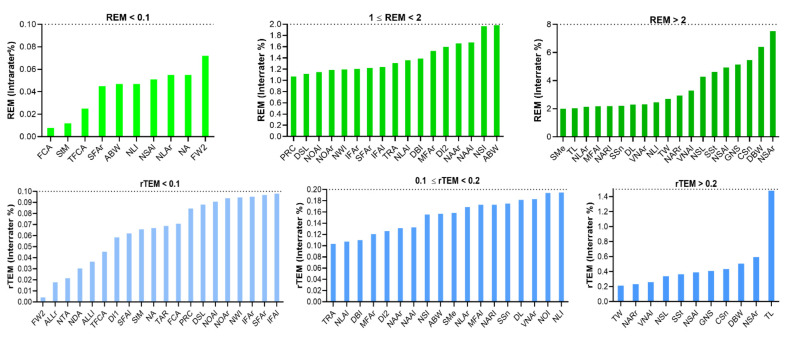
Inter-assessor relative error measurement (REM) and relative technical error of measurement (rTEM) of novel nasal measurements on three-dimensional images.

For novel measurements, 18 parameters had ICC values greater than or equal to 0.9 (excellent), and 25 parameters had ICC values between 0.80 and 0.89, indicating good reliability. The ICCs of TW, NLAl, IFAL, NDA, and CSn ranged from 0.75 to 0.79. All measurements showed good reliability, with ICCs above 0.75, except for TL, which was 0.73. The MAD of surface distance were 0.803 and 0.513 mm for GNS and DSL, respectively. The MAD of most linear distances were less than 1 mm. Only two parameters had an MAD slightly greater than 1 mm. The MAD were less than 1 degree for most angular parameters except VNAr, VNAl, and FCA. The REMs of 13 parameters were excellent and less than 1%. A total of 29 parameters had a REM between 1 and 3.9% (very good), while six parameters had a REM between 4 and 7% (good). The NSAr had the highest REM, with 7.522% (moderate). The rTEM was excellent for all parameters (<2%), with a maximum of 1.45% for TL (Table 6, Figure 8).

### 3.4. Intra-Method Reliability with VECTRA XT 3D Imaging System

ICCs were excellent across classical and novel measurements; all parameters were greater than or equal to 0.95 (Table 7 and Table 8). 

**Figure 9:**
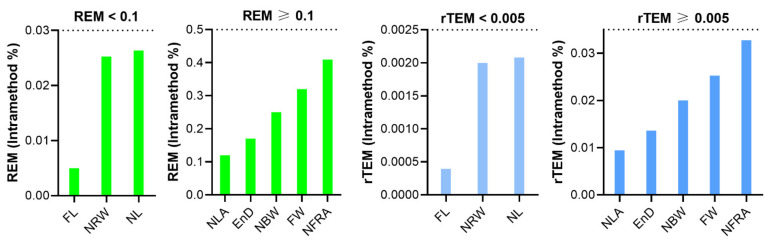
Intra-method relative error measurement (REM) and relative technical error of measurement (rTEM) of classic nasal measurements on three-dimensional images.

For classical measurements, the MAD was less than 0.3 mm for all linear distances, and 0.598 and 0.145 degrees for the angles. The REM were less than 0.5% and the rTEM were less than 0.04% for all parameters (excellent) (Table 7, Figure 9).

For novel measurements, the MAD of surface distance, GNS and DSL were 0.05 and 0.379 mm, respectively. Similar to intra-assessor reliability, the MAD was less than 0.2 degrees for all angular parameters. For the ratios, all MADs were less than 0.01 and the largest MAD value was 0.006 for NARI.

**Figure 10:**
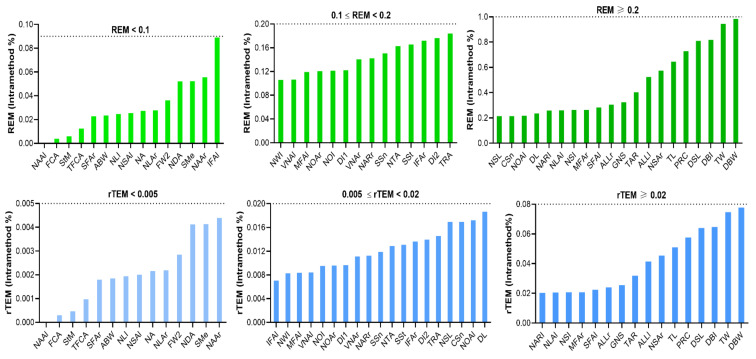
Intra-method relative error measurement (REM) and relative technical error of measurement (rTEM) of novel nasal measurements on three-dimensional images.

The REM of all parameters were less than 1%. A total of 15 parameters had a REM of less than 0.1% and 13 parameters had a REM between 0.1 and 0.2%. The remaining 21 parameters had a REM greater than 0.2%, with DBW having the largest REM at 0.9%. The rTEM was less than 0.1% for all parameters in the excellent category. The rTEM was less than 0.005% for 14 parameters, between 0.005 and 0.02% for 19 parameters, and above 0.02% for 16 parameters (Table 8, Figure 10). 

## 4. Discussion

This study assessed the accuracy and reliability of nasal anthropometry derived from 3D stereophotogrammetry. We introduced 46 novel and conventional 3D landmarks, as well as 57 corresponding novel and classical linear and surface distances and angular and ratio parameters for the quantitative analysis of perinasal morphometric parameters. These landmarks and parameters provide complete coverage of the nose and perinasal surface. 

The mean values of the measurements ranged from 6.264 to 186.334 mm for distance parameters, 23.824° to 174.779° for angular parameters, and from 0.179 to 2.437 for ratios. A very high level of agreement was found for intra-assessor reliability, with ICCs above 0.9 for 42 of the 57 parameters and above 0.8 for all parameters except NOAI. Furthermore, the validation results of the intra-assessor reliability showed that the REM (<1%) for almost all parameters and the rTEM (<1.5%) for all parameters were in the excellent category. 

For inter-assessor reliability, 42 of the 57 parameters were greater than 0.85, and 50 parameters were greater than 0.8. The results showed that inter-assessor reliability was slightly lower than the intra-assessor reliability, suggesting individual bias in the placement of landmarks despite the same workflow [22]. In terms of MAD, the most significant differences were 2.013 mm for the distance parameter, 1.471° for the angle parameter, and 0.065 for the ratio parameter. However, their correspondent REM were less than 3.9%, which is in the good category. We suggested that the main reason for the relatively significant MAD of these parameters is their own larger measured values. In terms of the rTEM, all parameters were less than 1%, except for TL (1.458%). Even so, the rTEMs for all parameters were in the excellent category. These findings suggest that despite the slight deviations in the locating of landmarks, measurements of inter-assessor reliability have proven to be highly consistent and reliable.

For intra-method reliability, the ICC was above 0.95 for most measurements, except for the NOAI of 0.948. The superb results of the intra-method assessment demonstrated the high reliability of the 3D imaging system. Considering the intra- and inter-assessor reliability, the landmark determination and placement protocol has been thoroughly evaluated and provides an effective and valid reference for further comparative and clinical research.

For the comparison of classic and novel measurements, most introduced novel nasal anthropometric parameters perform as reliably as the classical parameters. In terms of intra-assessor reliability, the REM of 42 novel parameters were in the excellent category and seven showed very good reliability. In terms of inter-assessor reliability, 42 novel parameters had reliability above the very good category, and six showed good reliability. In terms of intra-method reliability, all 49 novel parameters were in the excellent category. The rTEM of all novel parameters showed excellent reliability across intra- and inter-assessor and intra-method reliability. The largest deviations were concentrated around the nasal tip and nostrils. The reason for this might be the lack of consensual definition of the nasal tip boundary and the nostril short axis on 3D images. These resulted in the variation in the identification of the tip defining points and nostril short axis by different assessors. Nevertheless, almost all parameters displayed good reliability in the intra- and inter-assessor as well as the intra-method validation.

Our study demonstrated the excellent reliability of a novel 3D derived nasal anthropometry as well as the landmark-based setting approach. The results showed that most landmarks on 3D images obtained with the VECTRA XT, as well as the distances and angles between landmarks, are highly reliable. Reliability is one of the most commonly used indicators to assess the errors arising from a novel measurement process. It refers to the overall consistency of a measurement. The measurement is highly reliable if it produces similar results under consistent conditions or is consistent from one test occasion to another [23]. In this study, we implemented the five most frequently used estimates (MAD, REM, TEM, rTEM, ICC) based on previous studies to evaluate the avoidance of terminological confusion and make it easier for the reader to understand [11,18,19,24]. 

Although there have been some previous studies on nasal anthropometry using 3DSI, the facial landmarks and measurement parameters included are far from adequate for normal clinical practice. In particular, researchers did not perform the angle measurement and the linear measurement of the nasal tip area, and they similarly ignored the proportional relationship between the nose and the entire face [8,9,10,15,25]. To achieve harmony through invasive and non-invasive procedures, it is often necessary to correct the disproportions. The proportions of the face are of inestimable value when assessing the patient’s facial profile in consultations, as well as in surgical planning and assessment [26]. For this reason, we have attempted to fill this gap by introducing a richer set of facial landmarks and more detailed measurement parameters in order to provide a more comprehensive and objective reference. They need to be rigorously validated before being widely used in clinical practice and relevant research. In the current study, our results show that the newly defined facial landmarks and all measurement parameters used are sufficiently reliable to be used in clinical nasal anthropometry or basic research, especially for personalized rhinoplasty, consultation, as well as the design of maxillofacial surgery and pre- and post-operative follow-up.

In recent years, personalized medicine has become an increasingly popular conception [27,28]. Beauty seekers also demand a more detailed and all-sided level of personalized plastic surgery. The main drawback for patients undergoing traditional rhinoplasty is that the post-operative results are far from what is expected. Currently, the implant materials commonly used in rhinoplasty are manufactured in a standard mold and then sculpted by the surgeon on the operating table to match the external shape of the patient’s nose. The surgical outcome depends more on the aesthetics, experience, and skill level of the surgeon, and many patients do not realize until after the operation that the result is far from what they expected and have to remove the implant again [29,30]. With the introduction and popularity of 3DSI, digitally personalized plastic surgery has become possible. The facial landmarks and nasal anthropometric parameters involved in this study can provide plastic surgeons with more accurate and precise data on the patient’s nasal morphology, providing a more quantitative and objective reference for implant design and customization. It also allows an accurate comparison of changes in the patient’s pre- and post-operative nasal morphology. Furthermore, these 3DSI-based measurements combined with 3D printing technology can be used to personalize the design of the implant to better match the patient’s nasal morphology and avoid intra-operative re-sculpting, thereby significantly reducing the surgery time and alleviating the patients’ discomfort. 

## 5. Limitations and Perspectives

There were also some limitations in our study. Despite manual soft-tissue landmark placement in addition to automated landmark placement with the Vectra software, assessor-dependent errors could not be completely excluded. In this regard, we believe that, for those landmarks that involve relatively less reliability, such as TDP, NOAr, and NOAl, assessors can mark these points on the face manually in advance to reduce the assessor-dependent error and obtain more objective and accurate results. 

In our future research, we will recruit various groups of participants, evaluating the method’s reliability in subjects in wider age ranges and in different races. Moreover, we will use these facial landmarks and anthropometric parameters to compare the differences in nasal morphology between ethnic groups.

## 6. Conclusions

This research introduces 46 facial landmarks and 57 detailed three-dimensional digital nasal and perinasal anthropometric parameters, demonstrating their high reliability for the analysis of nasal morphological features. It offers essential evidence and an initial reference for the application of 3D nasal anthropometry in clinical practice. Compared to previous studies in the oral and maxillofacial area on mannequins and nasal or morphology analysis based on 3DSI, our study included much more comprehensive and detailed anthropometric parameters. This provides clinicians with a more comprehensive and extensive range of nasal morphological data. As the first study we know of using 3D stereophotography to evaluate the reliability of nose measurements in detail, this study could be the primary foundation for this field. This technology can be used for surgical planning and the evaluation of post-operative effects in the field of otolaryngology, plastic and cosmetic surgery, and maxillofacial surgery that seeks to change the nasal morphology.

## Figures and Tables

**Table 1:** Demographic characteristics of study participants.

Age	Males (*n* = 55)	Females (*n* = 55)
Mean ± SD	42.23 ± 8.31	40.53 ± 7.99
Range	18–62 yrs	18–65 yrs
18–25	9	11
26–35	13	13
36–45	14	12
46–55	12	10
≥55	7	9

**Table 2:** The name and definitions of 46 anthropometric nasal landmarks involved in this study.

Classification	Landmarks	Definition
Novel	Nostril base point left (Nb l)	the lowest point of each nostril or the inferior terminal point of left nostril axis.
	Nostril base point right (Nb r)	the lowest point of each nostril or the inferior terminal point of right nostril axis.
	Nostril lateral point left (Nl l)	the junction point of nostril short axis and the lateral margin of left nostril
	Nostril lateral point right (Nl r)	the junction point of nostril short axis and the lateral margin of right nostril
	Nostril medial point right (Nm l)	the junction point of nostril short axis and the medial margin of left nostril
	Nostril medial point right (Nm r)	the junction point of nostril short axis and the medial margin of right nostril
	Nostril top points left (Nt l)	the highest point of each nostril or the superior terminal point of left nostril axis.
	Nostril top points right (Nt r)	the highest point of each nostril or the superior terminal point of right nostril axis.
	Sellion’ left (Se’ l)	the left intersections of TH[Se] and Dorsal aesthetic lines
	Sellion’ right (Se’ r)	the right intersections of TH[Se] and Dorsal aesthetic lines
	Highnasal (Hn) or Lownasal(Ln)	the most anterior or posterior point on dorsum of nose between its root and tip
	Sublabiale(Sl)	the most posterior midpoint on the labiomental soft tissue contour that defines the border between the lower lip and the chin.
	Supramental (Sm)	Deepest point in inferior sublabial concavity
	Columella constructed point (Cc)	the midpoint of the columella crest at the level of the nostril top points
	Ort left	the left Junction of true vertical (TV) and true horizontal (TH) on the alare
	Ort right	the right Junction of true vertical (TV) and true horizontal (TH) on the alare
	Supratip break (Stb)	the joint point of the dorsum and nasal tip
	Postaurale left (Pa l)	Most posterior point on the free margin of the left ear
	Postaurale right (Pa r)	Most posterior point on the free margin of the right ear
Classic	Em left	Lower margin of the left medial eyebrow end
	Em right	Lower margin of the right medial eyebrow end
	Endocanthion left (En l)	the left inner commissure of the palpebral fissure, the rightmidpoint of the frontonasal suture
	Endocanthion right (En r)	the right inner commissure of the palpebral fissure, the rightmidpoint of the frontonasal suture
	Alar curvature/Alar crest left (Ac l)	Alar curvature point (ac) is the point located at the facial insertion of left alar base.
	Alar curvature/Alar crest right (Ac r)	Alar curvature point (ac) is the point located at the facial insertion of right alar base.
	Alare left (Al l)	the most lateral point on left alar contour
	Alare right (Al r)	the most lateral point on right alar contour
	Columella (Cm)	Most anterior and inferior point on apex of nose
	Glabella (G)	Most anterior point on midline of forehead
	Nasion (N)	Deepest point in middle of frontonasal curve
	Pronasale (Prn)	Most prominent point on apex of nose
	Sellion (Se)	the most posterior point of the sagittal plane Sin the midline of the nasal root.
	Subnasale (Sn)	Deepest point in nasolabial curvature
	Tip defining point left(TDP l)	the left most anterior projection of the tip cartilage
	Tip defining point right (TDP r)	the right most anterior projection of the tip cartilages
	Cervical (C)	Deepest point at angel of chin and neck
	Labrale inferius (Li)	Lower lip vermilion border
	Labrale superius (Ls)	Upper lip vermilion border
	Menton (Me)	Most inferior point on inferior edge of chin
	Stomion(Sto)	the midpoint of the horizontal labial fissure
	Pogonion (Pg)	Most anterior midpoint of chin
	Tragus left (Trg l)	Most posterior point of auricular tragus left
	Tragus right (Trg r)	Most posterior point of auricular tragus right
	Trichion (Tri)	Intersection of hairline and midline of forehead
	Zygion left (Zy l)	the most lateral point on the outline of left zygomatic arch
	Zygion right (Zy r)	the most lateral point on the outline of right zygomatic arch

**Table 3:** List of 57 nasal anthropometric parameters.

Classification	Measurements	Abbreviation	Landmarks or Definitions
Classic	Projective linear (Straight line) distance		
	Face width	FW	Zy(l)-Zy(r)
	Face length	FL	Tri-Me
	Nasal root width	NRW	Em(l)-Em(r)
	Inner intercanthal length	EnD	En (l)-En (r)
	Nasal length	NL	G-Sn
	Nasal base width	NBW	Ac(l)-Ac(r)
	Angles		
	Nasalfrontal Angel	NFRA	G-Se-Prn
	Nasolabial Angle	NLA	Cm-Sn-Ls
Novel	Surface linear distance		
	Glabella-Nasion-Sellion	GNS	G-N-Se
	Dorsum surface length	DSL	Se-stp-prn
	Projective linear dimensions (Straight line distance)		
	Face width2	FW2	Postaurale(l)- Postaurale(r)
	Sellion-Subnasal	SSn	Se-Sn
	Dorsal bridge width(narrowest)	DBW	Se’(l)-Se’(r)
	Nasion-Alare *	NAL	N-Al
	Dorsum length	DL	Se-Prn
	Alar base width	ABW	Al(l)-Al(r)
	Alare length	ALL	Prn-Ac
	Tip width	TW	TDP(l)-TDP(r)
	Tip length	TL	Stb-Cm
	Nasal Septum length	NSL	Cm-Sn
	Pronasale-Columella	PRC	Prn-Cm
	Nostril lang Axis length *	NLA	Nt-Nb
	Nostril short Axis length *	NSA	shortest distance perpendicular to NLA
	Subnasal-Stomion	SSt	Sn-Sto
	Subnasal-Menton	SMe	Sn-Me
	Stomion-Menton	StM	St-Me
	Columella-Subnasal	CSn	Cm-Sn
	Angles		
	Nasal Dorsum Angle	NDA	Se-Hn-Prn/Se-Ln-Prn
	Vertical nasal angle	VNA	Prn-Se-Ort
	Nasal Angel	NA	Sn-Prn-Se
	Superior facial third Angle	SFA	Tri-Trg-Se
	Middle facial third Angle	MFA	Se-Trg-Sn
	Inferior facial third Angle	IFA	Sn-Trg-Me
	Total facial convexity Angel	TFCA	G-Prn-Pg
	Facial convexity Angel	FCA	G-Sn-Pg
	Tip rotation Angel	TRA	180-(Prn-Cm-Sn)
	Nostril Angel *	NOA	NtR-NbR-Sn
	Nasal Tip Angel	NTA	Stb-Prn-Sn
	Ratio		
	Nasal width Index	NWI	NBW/FW2
	Nasal length Index	NLI	NL/FL
	Dorsum Index-1	DI-1	SSn/NL
	Dorsum Index-2	DI-2	SSn/Stm
	Nasolabial Index	NOI	SSt/SMe
	Dorsal bridge Index	DBI	DBW/EnD
	Tip Aspect ratio	TAR	TW/TL
	Nostril Aspect ratio *	NAR	NLA/NSA
	Nasal Septum Index *	NSI	NSL/NSL+PRC

* Both sides.

**Table 4:** Summary of reliability estimates evaluated.

Statistics	Equation
Intraclass correlation coefficient (ICC)	B/(B + W)
Mean absolute difference (MAD)	|X1 − X2|
Relative error measurement (REM)	(MAD/X3) × 100
Technical error of measurement (TEM)	$\sqrt{(\sum D^{2}/2N})$
Relative TEM (rTEM)	(TEM/X3) × 100

B, between-measurement variance. W, within-measurement variance; D, difference between measurements. N, number of subjects measured. X1, mean for assessor 1 (session 1, or session 2 of capture 1). X2, mean for assessor 2 (session 2, or session 2 of capture 2); X3, grand mean.

**Table 5:** The intra-assessor and inter-assessor reliability of classic parameters.

		Intra-Assessor					Inter-Rater				
Classification	Variable	MAD	REM (%)	TEM	rTEM(%)	ICC	MAD	REM (%)	TEM	rTEM (%)	ICC
Linear Distance	FW	***0.758***	0.641	0.06	0.051	0.96	**1.791**	**1.502**	0.142	0.119	0.90
	FL	0.019	0.01	0.001	0.001	0.99	0.364	0.195	0.029	0.015	0.98
	NRW	0.013	0.051	0.001	0.004	0.97	0.694	**2.69**	0.055	0.213	0.94
	EnD	0.101	0.344	0.008	0.027	0.92	0.003	0.01	0	0.001	0.81
	NL	0.035	0.053	0.003	0.004	0.96	**2.013**	**2.975**	0.159	0.235	0.81
	NBW	0.155	0.506	0.012	0.04	0.96	0.15	0.49	0.012	0.039	0.85
Angles	NFRA	0.044	0.03	0.003	0.002	0.99	0.397	0.275	0.031	0.022	0.97
	NLA	0.29	0.239	0.023	0.019	0.98	0.993	0.82	0.065	0.054	0.95

The bolded values are referred to in main text in Results.

**Table 6:** The intra-assessor and inter-assessor reliability of novel parameters.

		Intra-Assessor					Inter-Rater				
Classification	Variable	MAD	REM (%)	TEM	rTEM (%)	ICC	MAD	REM (%)	TEM	rTEM (%)	ICC
Surface distance	GNS	0.1	0.647	0.008	0.051	0.86	0.808	5.155	0.064	0.408	0.88
	DSL	0.216	0.473	0.017	0.037	0.98	0.513	1.115	0.041	0.088	0.89
Linear distance	FW2	0.124	0.072	0.01	0.006	0.99	0.087	0.051	0.007	0.004	0.83
	SSn	0.156	0.301	0.012	0.024	0.96	1.158	2.213	0.092	0.175	0.88
	DBW	0.286	**1.958**	0.023	0.155	0.91	0.906	6.395	0.072	0.506	0.89
	NAAr	0.058	0.111	0.005	0.009	0.97	0.878	1.661	0.069	0.131	0.88
	NAAl	0.173	0.33	0.014	0.026	0.96	0.885	1.677	0.07	0.133	0.86
	DL	0.228	0.452	0.016	0.036	0.98	1.063	2.296	0.084	0.182	0.88
	ABW	0.014	0.047	0.001	0.004	0.97	0.61	1.986	0.048	0.157	0.97
	ALLr	0.193	0.609	0.015	0.048	0.94	0.071	0.224	0.006	0.018	0.92
	ALLl	**0.335**	1.045	0.026	0.083	0.93	0.147	0.461	0.012	0.036	0.91
	TW	0.188	1.881	0.015	0.149	0.87	0.274	2.702	0.022	0.214	0.76
	TL	0.139	1.286	0.011	0.102	0.9	0.139	2.041	0.161	1.481	**0.73**
	NSL	0.065	0.429	0.005	0.034	0.88	0.662	4.292	0.052	0.339	0.92
	PRC	0.091	1.452	0.007	0.115	0.82	0.067	1.069	0.005	0.085	0.86
	NLAr	0.008	0.055	0.001	0.004	0.97	0.319	2.135	0.025	0.169	0.79
	NLAl	0.078	0.52	0.006	0.041	0.96	0.205	1.358	0.016	0.107	0.97
	NSAr	0.077	1.152	0.006	0.091	0.98	0.52	7.522	0.041	0.595	0.94
	NSAl	0.003	0.051	0	0.004	0.96	0.317	4.933	0.025	0.39	0.86
	SSt	0.072	0.331	0.006	0.026	0.89	0.979	4.62	0.077	0.365	0.81
	SMe	0.072	0.105	0.006	0.008	0.96	1.373	2.004	0.109	0.158	0.85
	StM	0.006	0.012	0	0.001	0.95	0.395	0.831	0.031	0.066	0.86
	CSn	0.065	0.429	0.005	0.034	0.88	0.849	5.474	0.067	0.433	0.79
Angles	NDA	0.182	0.104	0.014	0.008	0.86	0.669	0.384	0.053	0.03	0.77
	VNAr	0.124	0.281	0.01	0.022	0.94	1.009	2.316	0.08	0.183	0.81
	VNAl	0.092	0.213	0.007	0.017	0.91	1.4	3.293	0.111	0.26	0.83
	NA	0.053	0.055	0.004	0.004	0.96	0.828	0.846	0.065	0.067	0.95
	SFAr	0.014	0.045	0.001	0.004	0.98	0.362	1.224	0.029	0.097	0.93
	SFAl	0.169	0.565	0.013	0.045	0.98	0.234	0.785	0.019	0.062	0.92
	MFAr	0.125	0.527	0.01	0.042	0.86	0.366	1.524	0.029	0.12	0.90
	MFAl	0.051	0.212	0.004	0.017	0.93	0.534	2.186	0.042	0.173	0.86
	IFAr	0.1	0.345	0.008	0.027	0.92	0.346	1.206	0.027	0.095	0.80
	IFAl	0.052	0.178	0.004	0.014	0.94	0.359	1.24	0.028	0.098	0.76
	TFCA	0.034	0.025	0.003	0.002	**1**	0.789	0.574	0.062	0.045	0.99
	FCA	0.012	0.008	0.001	0.001	0.98	1.471	0.895	0.116	0.071	0.96
	TRA	0.136	0.368	0.011	0.029	0.87	0.479	1.308	0.038	0.103	0.84
	NOAr	0.121	0.243	0.01	0.019	0.81	0.595	1.187	0.047	0.094	0.91
	NOAl	0.206	0.435	0.016	0.034	**0.77**	0.546	1.148	0.043	0.091	0.94
	NTA	0.258	0.325	0.02	0.026	0.93	0.216	0.272	0.017	0.021	0.93
Ratio	NWI	0.001	0.556	0	0.111	0.96	0.002	1.197	0	0.095	0.86
	NLI	0	0.047	0	0.004	0.95	0.009	2.464	0.001	0.195	0.81
	DI1	0.002	0.243	0	0.019	0.86	0.004	0.516	0	0.058	0.86
	DI2	0.004	0.352	0	0.028	0.95	0.018	1.596	0.001	0.126	0.94
	NOI	0.001	0.242	0	0.019	0.87	0.003	0.962	0.001	0.194	0.84
	DBI	0.008	1.627	0.001	0.129	0.85	0.007	1.391	0.001	0.11	0.91
	TAR	0.008	0.803	0.001	0.064	0.81	0.008	0.87	0.001	0.069	0.97
	NARr	0.006	0.284	0.001	0.022	0.98	0.065	2.939	0.005	0.232	0.92
	NARI	0.013	0.516	0.001	0.04	0.98	0.053	2.187	0.004	0.173	0.98
	NSI	0.004	0.526	0	0.04	0.83	0.014	1.966	0.001	0.155	0.82

The bolded values are referred to in main text in Results.

**Table 7:** The intra-method reliability of classic parameters.

Classification	Variable	Capture 1	Capture 2	MAD	REM	TEM	rTEM(%)	ICC
Linear Distance	FW	118.33	118.71	0.379	0.32	0.03	0.025	0.99
	FL	186.33	186.34	0.009	0.01	0.001	0	1
	NRW	26.15	26.15	0.007	0.03	0.001	0.002	0.99
	EnD	29.29	29.24	0.05	0.17	0.004	0.014	0.98
	NL	66.67	66.69	0.018	0.03	0.001	0.002	0.99
	NBW	30.59	30.51	0.077	0.25	0.006	0.02	1
Angles	NFRA	144.08	144.67	0.598	0.41	0.047	0.033	1
	NLA	121.58	121.73	0.145	0.12	0.011	0.009	1

**Table 8:** The intra-method reliability of novel parameters.

Classification	Variable	Capture 1	Capture 2	MAD	REM	TEM	rTEM(%)	ICC
Surface distance	GNS	15.46	15.41	0.05	0.32	0.004	0.026	0.97
	DSL	46.58	46.96	0.379	0.81	0.03	0.064	0.99
Linear Distance	FW2	171.43	171.36	0.062	0.04	0.005	0.003	1
	SSn	51.75	51.83	0.078	0.15	0.006	0.012	0.99
	DBW	14.62	14.48	0.143	0.98	0.011	0.078	0.98
	NAAr	52.41	52.44	0.029	0.06	0.002	0.004	0.99
	NAAl	52.33	52.33	0	0	0	0	0.99
	DL	45.77	45.88	0.108	0.24	0.009	0.019	0.99
	ABW	30.43	30.42	0.007	0.02	0.001	0.002	0.99
	ALLr	31.72	31.62	0.097	0.31	0.008	0.024	0.99
	ALLl	32.04	31.88	0.167	0.52	0.013	0.041	0.98
	TW	10.01	9.92	0.094	0.95	0.007	0.075	0.97
	TL	10.82	10.76	0.07	0.65	0.006	0.051	0.98
	NSL	15.09	15.13	0.032	0.21	0.003	0.017	0.97
	PRC	6.26	6.22	0.045	0.73	0.004	0.058	0.95
	NLAr	14.8	14.8	0.004	0.03	0	0.002	0.99
	NLAl	15	15.04	0.039	0.26	0.003	0.021	0.99
	NSAr	6.66	6.69	0.038	0.58	0.003	0.045	0.99
	NSAl	6.28	6.28	0.002	0.03	0	0.002	0.99
	SSt	21.68	21.71	0.036	0.17	0.003	0.013	0.97
	SMe	69.2	69.24	0.036	0.05	0.003	0.004	0.99
	StM	47.77	47.76	0.003	0.01	0	0	0.99
	CSn	15.09	15.13	0.032	0.21	0.003	0.017	0.97
Angles	NDA	174.78	174.87	0.091	0.05	0.007	0.004	0.97
	VNAr	44.08	44.02	0.062	0.14	0.005	0.011	0.98
	VNAl	43.2	43.15	0.046	0.11	0.004	0.008	0.98
	NA	97.4	97.37	0.027	0.03	0.002	0.002	0.99
	SFAr	29.78	29.78	0.007	0.02	0.001	0.002	1
	SFAl	29.96	29.88	0.085	0.28	0.007	0.022	1
	MFAr	23.82	23.89	0.063	0.26	0.005	0.021	0.97
	MFAl	24.14	24.16	0.026	0.11	0.002	0.008	0.98
	IFAr	28.91	28.96	0.05	0.17	0.004	0.014	0.98
	IFAl	29.11	29.14	0.026	0.09	0.002	0.007	0.99
	TFCA	136.92	136.9	0.017	0.01	0.001	0.001	1
	FCA	163.64	163.65	0.006	0	0	0	1
	TRA	36.85	36.78	0.068	0.18	0.005	0.015	0.97
	NOAr	49.8	49.74	0.06	0.12	0.005	0.01	0.95
	NOAl	47.34	47.24	0.103	0.22	0.008	0.017	0.95
	NTA	79.45	79.32	0.129	0.16	0.01	0.013	0.98
Ratio	NWI	0.18	0.18	0	0.11	0	0.008	0.98
	NLI	0.36	0.36	0	0.03	0	0.002	0.98
	DI1	0.78	0.78	0.001	0.12	0	0.01	0.96
	DI2	1.09	1.09	0.002	0.18	0	0.014	0.99
	NOI	0.31	0.31	0	0.12	0	0.01	0.96
	DBI	0.5	0.5	0.004	0.82	0	0.065	0.97
	TAR	0.94	0.93	0.004	0.4	0	0.032	0.95
	NARr	2.26	2.26	0.003	0.14	0	0.011	0.99
	NARI	2.43	2.44	0.006	0.26	0	0.02	0.99
	NSI	0.71	0.71	0.002	0.26	0	0.021	0.96

## Data Availability

The data are not publicly available due to the protection of patients’ privacy and image rights.

## Supplementary Materials

The following are available online at https://www.mdpi.com/article/10.3390/jpm12010060/s1, Table S1: Means and SDs (mm or degree) across all measurement categories of two assessors.
