# Supplementary material for: Investigating the Reliability of Novel Nasal Anthropometry Using Advanced Three-Dimensional Digital Stereophotogrammetry
Source: J Pers Med. 2022 Jan 6;12(1):60. doi: 10.3390/jpm12010060 (PMC8777712; doi:10.3390/jpm12010060)
Supplement: Supplementary file 1 [file jpm-12-00060-s001.zip › jpm-1502051-supplementary.pdf]

Supplementary Table S1. Means and SDs (mm or degree) across all measurements of two assessors

Classifications	Parameters	Assessor 1				Grand Mean	Intra-assessor P value	Assessor 2		Inter-assessor P value
		Measurement session1		Measurement session2				Measurement		
		μ	SD	μ	SD			μ	SD	
Classic	FW	117.95	8.29	118.71	5.81	118.33	0.62	120.12	6.07	<0.01*
	FL	186.33	10.14	186.34	10.07	186.33	0.93	186.70	9.75	0.29
	NRW	26.16	4.75	26.15	4.39	26.15	0.94	25.46	4.56	0.01
	EnD	29.34	2.33	29.24	2.19	29.29	0.47	29.29	2.50	0.99
	NL	66.65	4.24	66.69	4.36	66.67	0.85	68.68	4.71	0.08
	NBW	30.67	3.00	30.51	3.05	30.59	0.25	30.44	3.77	0.59
	NFRA	144.05	6.52	144.10	6.41	144.07	0.76	144.47	6.18	0.09
	NLA	121.44	9.54	121.73	9.55	121.58	0.29	120.59	9.91	0.11
Novel	GNS	15.51	2.59	15.41	2.33	15.46	0.23	15.90	2.69	0.26
	DSL	46.54	4.25	46.63	4.18	45.77	0.13	46.29	3.12	0.07
	FW2	171.49	12.24	171.36	12.70	171.43	0.59	171.34	12.73	0.47
	SSn	51.67	3.71	51.83	3.78	51.75	0.32	52.91	3.94	0.06
	DBW	14.77	2.45	14.48	2.61	14.62	0.08	13.72	2.66	<0.01*
	NALr	52.38	3.86	52.44	3.88	52.41	0.71	53.29	3.52	0.11
	NALl	52.25	3.87	52.42	3.76	52.33	0.32	53.22	3.90	0.08
	DL	45.66	4.05	45.88	4.09	45.77	0.13	46.84	3.84	0.06
	ABW	30.44	3.47	30.42	3.49	30.43	0.91	31.04	3.24	0.07
	ALLr	31.82	2.74	31.62	3.02	31.72	0.21	31.79	2.97	0.69
	ALLl	32.21	2.67	31.88	3.00	32.04	0.04	31.90	2.97	0.42
	TW	10.11	1.50	9.92	1.47	10.01	0.09	10.29	1.64	0.17
	TL	10.89	1.55	10.76	1.49	10.82	0.18	10.96	1.92	0.43
	NSL	15.06	1.93	15.13	1.89	15.09	0.64	15.76	2.17	0.22
	PRC	6.31	1.02	6.22	0.91	6.26	0.29	6.20	0.86	0.33
	NLAr	14.81	1.95	14.80	1.95	14.80	0.91	15.12	2.77	0.16
	NLAl	14.96	2.10	15.04	2.17	15.00	0.39	15.20	2.20	0.12
	NSAr	6.62	1.20	6.69	1.19	6.65	0.07	7.18	1.23	0.06
	NSAl	6.28	1.19	6.28	1.08	6.28	0.95	6.59	1.14	0.13
	SSt	21.64	2.22	21.71	2.19	21.68	0.65	20.70	2.42	<0.01*
	SMe	69.16	5.51	69.24	5.52	69.20	0.78	67.83	5.07	0.19
	StM	47.77	4.37	47.76	4.41	47.76	0.98	47.37	4.17	0.24
	CSn	15.06	1.93	15.13	1.89	15.09	0.64	15.94	2.83	0.33
	NDA	174.69	3.02	174.87	3.19	174.78	0.45	174.11	3.40	0.08
	VNAr	44.15	3.50	44.02	3.28	44.08	0.50	43.08	4.41	0.27
	VNAl	43.24	3.42	43.15	3.26	43.20	0.67	41.80	4.42	0.34
	NA	97.43	4.49	97.37	4.60	97.40	0.79	98.23	4.55	0.09
	SFAr	29.79	4.00	29.78	3.90	29.78	0.91	29.42	3.94	0.11
	SFAl	30.05	3.87	29.88	3.89	29.96	0.14	29.73	3.91	0.33
	MFAr	23.76	1.55	23.89	1.41	23.82	0.28	24.19	1.49	0.07
	MFAl	24.11	1.54	24.16	1.51	24.14	0.56	24.67	1.55	0.22
	IFAr	28.86	2.49	28.96	2.49	28.91	0.51	28.56	2.52	0.06
	IFAl	29.09	2.36	29.14	2.49	29.11	0.70	28.75	2.47	0.13
	TFCA	136.94	5.23	136.90	5.15	136.92	0.68	137.71	5.19	0.31
	FCA	163.64	5.39	163.65	5.61	163.64	0.95	165.11	5.20	0.09
	TRA	36.91	7.48	36.78	6.86	36.85	0.81	36.37	6.72	0.39
	NOAr	49.86	7.96	49.74	6.84	49.80	0.86	50.39	6.64	0.17
	NOAl	47.44	7.75	47.24	8.22	47.34	0.79	47.89	7.60	0.30
	NTA	79.58	8.02	79.32	7.95	79.45	0.58	79.24	7.76	0.62
	NWI	0.18	0.02	0.18	0.02	0.18	0.60	0.18	0.02	0.28
	NLI	0.36	0.02	0.36	0.02	0.36	0.88	0.37	0.02	0.04
	DI1	0.78	0.03	0.78	0.03	0.78	0.47	0.77	0.03	0.30
	DI2	1.09	0.11	1.09	0.11	1.09	0.49	1.11	0.11	0.01
	NOI	0.31	0.02	0.31	0.02	0.31	0.67	0.31	0.03	0.09
	DBI	0.50	0.08	0.50	0.08	0.50	0.22	0.49	0.08	0.17
	TAR	0.94	0.15	0.93	0.14	0.93	0.55	0.94	0.13	0.42
	NARr	2.26	0.37	2.26	0.38	2.26	0.62	2.20	0.31	0.11
	NARl	2.42	0.44	2.44	0.43	2.43	0.40	2.38	0.44	0.09
	NSI	0.70	0.04	0.71	0.04	0.71	0.27	0.72	0.04	0.11

****P<0.05***
